# Aspirin suppresses growth of human gastric carcinoma cell by inhibiting survivin expression^☆^

**DOI:** 10.1016/S1674-8301(11)60033-X

**Published:** 2011-07

**Authors:** Li Yang, Huaijun Zhu, Dongxiao Liu, Song Liang, Hao Xu, Jie Chen, Xuerong Wang, Zekuan Xu

**Affiliations:** a Department of General Surgery, the First Affiliated Hospital of Nanjing Medical University, Nanjing, Jiangsu 210029, China;; b Division of Clinical Pharmacy, Department of Pharmacy, Drum Tower Hospital Affiliated to Medical School of Nanjing University, Nanjing, Jiangsu 210008, China;; c Department of Pharmacology, Nanjing Medical University, Nanjing, Jiangsu 210029, China.

**Keywords:** aspirin, survivin, gastric cancer, apoptosis

## Abstract

Regular use of aspirin (ASA) could reduce the risk of gastric cancer although the precise mechanism remains unclear. Down-regulation of survivin may be one of the cyclooxygenase-independent mechanisms whereby ASA induces apoptosis of gastric cancer cell. In this study, we investigated the effect of ASA on the growth, apoptosis and survivin expression of gastric cancer cell line SGC7901. The survival of cells treated with 3.0 and 10.0 mmol/L ASA for 24 h was decreased by 44.6% and 88.5%, respectively. ASA at 3.0 mmol/L inhibited the viability of SGC7901 cells in a time-dependent manner. Apoptosis analysis showed similar results with MTT assay. ASA at 3.0 and 10.0 mmol/L decreased the mRNA transcript levels of *survivin* and reduced survivin protein levels in SGC7901 cells also in a time-dependent manner. Our findings indicated that ASA inhibited the proliferation of SGC7901 by suppressing survivin at both the transcriptional and translational level.

## INTRODUCTION

Non-steroidal antiinflammatory drugs (NSAIDs), such as aspirin (acetylsalicylic acid, ASA), are commonly used to relieve pain and reduce inflammation. In the past decade, accelerated studies have indicated the anticancer effects of ASA in a variety of organs including the stomach. However, the precise mechanism remains unknown[1]–[3]. ASA is known to directly inhibit the activity of cyclooxygenase 2 (COX-2) and consequently decrease the synthesis of prostaglandins E2 (PGE-2), resulting in apoptosis of cancer cells[1],[4]. However, recent studies suggested that the anticancer action of NSAIDs may also be mediated by COX-independent apoptosis[3],[5],[6], such as down-regulation of antiapoptotic proteins.

Survivin, a member of the inhibitor of apoptosis (IAP) family, is a unique bifunctional protein that potentially inhibits apoptosis and regulates cell division[7]. It is commonly overexpressed in a wide variety of human malignancies and plays an important role in the occurrence and progression of cancers including gastric cancer[8],[9]. Additionally, previous studies by us and others have confirmed that survivin expression in tumors is implicated in tumor resistance to chemotherapy and radiotherapy[10]–[13]. Thus, survivin has been indicated to be a promising target for cancer therapy and overcoming chemotherapy resistance.

Recently, ASA has been reported to mediate the COX-independent apoptosis by down regulating survivin in hormone-refractory prostate cancer cells and transformed breast epithelial cells. Yoo *et al*.[14] suggested that the down-regulation resulted from inhibition of E2F-1 binding to the survivin promoter region. Lu *et al*.[15] indicated that ASA reduced the levels of survivin by inducing its proteasomal degradation. Furthermore, by downregulating survivin, ASA was found to promote tumor necrosis factor-related apoptosis-inducing ligand (TRAIL) induced apoptosis. However, no study was reported on the association between ASA and survivin in gastric cancer.

Although the reduction of survivin by ASA is unproven in the stomach, other NSAIDs have been reported to show such an effect. Chiou *et al*.[16] found that the non-selective COX inhibitor indomethacin could reduce survivin expression in gastric mucosal cells, but this effect was not shown in selective COX-1 or COX-2 inhibitors as well as the combination of the two. The evidence indicated that some NSAIDs could suppress survivin expression in the stomach in a COX-independent manner. Given the clinical anticancer effect on the stomach and induction of apoptosis by down regulating survivin in other cells, ASA may also inhibit survivin in gastric cancer. In this study, we investigated the effect of ASA on growth, apoptosis and survivin expression of gastric cancer cell line SGC7901.

## MATERIALS AND METHODS

### Regents

Human gastric carcinoma SGC7901 cell line was provided by the Cell Bank of Shanghai Institute of Cell Biology, Chinese Academy of Sciences (Shanghai, China). Cells were cultured in RPMI 1640 (Invitrogen, CA, USA) supplemented with 10% fetal bovine serum (FBS, Hyclone, UT, USA), penicillin (100 U/mL) and streptomycin (0.1 g/L) at 37°C in a humidified atmosphere containing 5% CO_2_. ASA was purchased from Sigma-Aldrich (St. Louis, MO, USA) and dissolved in dimethyl sulfoxide (DMSO). ASA was prepared in culture medium and the concentration of DMSO in the medium was not more than 0.05%. Human anti-survivin and anti-actin antibodies were purchased from R&D systems (MN, USA).

### Trypan blue exclusion assay

Trypan blue exclusion assay was conducted to evaluate the proper concentration of ASA in this study. Briefly, trypsinized SGC7901 cells were pelleted, resuspended (5×10^4^/mL) and seeded in a 96-well plate (200 µL/well). After incubation for 24 h, the medium was aspirated and replaced with 200 µL of medium containing 0, 0.3, 1.0, 3.0, 10.0, and 30.0 mmol/L ASA. Cells incubated with medium containing 0.05% DMSO served as vehicle control. After incubation for an additional 24 h, cells were trypsinized and resuspended in 50 µL of medium and mixed with 50 µL of 0.4% trypan blue solution. The mixture was incubated at room temperature for 15 min and then examined under a light microscope. Photographs of 4 random views were taken for each sample and the percentage of stained cells was determined.

### Cell viability assay

Cell viability was determined by 3-(4,5-dimethylthiazol-2-yl)-2,5-diphenyltetrazolium bromide (MTT) reduction assay. SGC7901 cells were seeded in a 96-well plate (10^4^/well) and incubated for 24 h. In the dose-dependent experiment, cells were treated with vehicle (medium with 0.05% DMSO) and ASA (1.0, 3.0 and 10.0 mmol/L) for 24 h, while cells were treated with vehicle and 3 mmol/L ASA for 0, 24, 48 and 72 h in time-dependent experiment. Prior to measurement, a volume of 20 µL 0.5% MTT in phosphate buffered saline (PBS) was added to each well. After 4 h incubation, the culture media were discarded and 200 µL of DMSO were added followed by vibration for 10 min. The absorbance (*A*) was read at 490 nm with ELX 800 (Biokit, MA, USA). Cell survival rate was calculated as follows: *A*_ASA_/*A*_vehicle_×100%.

### Cell apoptosis assay

Apoptosis assay was conducted by flow cytometry using the Annexin V-FICT/propidium iodide apoptosis detection kit (BD Bioscience, USA) according to the manufacturer's instructions. After treatment with ASA (1.0, 3.0 and 10.0 mmol/L) for 24 h and 3.0 mmol/L ASA for 24, 48 and 72 h, SGC7901 cells were harvested, washed with PBS twice and then resuspended in 500 µL of binding buffer. A volume of 5 µL of Annexin V-FITC and 5 µL of propidium iodide were added and the mixture was incubated for 15 min in the dark. The apoptotic rates were determined by FACS Calibar (BD, CA, USA) and the data were analyzed by Modfit V 3.0.

### RT-PCR analysis of survivin mRNA

Total RNA was extracted from cells treated with ASA (1, 3 and 10 mmol/L) for 24 h and 3.0 mmol/L ASA for 24, 48 and 72 h, using the TRIzol method (Invitrogen, CA, USA) according to the manufacturer's instructions. cDNA was synthesized by RNA using the Superscript First-Strand Synthesis System for RT-PCR (Invitrogen). *Survivin* mRNA was amplified using the primers: sense, 5′-GCATGGGTGCCCCGACGTTG-3′, and antisense, 5′-GCTCCGGCCAGAGGCCTCAA-3′. *β*-*actin* mRNA was used as control and the primers were 5′-TAAAGACCTCTATGCCAACACAGT-3′ and 5′-CACCATGGAGGGGCCGGACTCTTC-3′. The PCR reaction was performed in a total volume of 20 µL containing 0.1 mmol/L dNTPs, 0.5 µmol/L of each primer, 1 U of *Taq* DNA polymerase (MBI Fermentas, Vilnius, Lithuania) and MgCl_2_ of 0.8 mmol/L for *survivin*, while 1.6 mmol/L for *β*-*actin*. The amplification conditions were as follows: *survivin*, 30 cycles of 94°C for 30 s, 55°C for 60 s and 72°C for 60 s, and *β*-*actin*, 28 cycles of 94°C for 30 s, 58°C for 40 s and 72°C for 40 s. The PCR products were separated on 1% agarose gel containing ethidium bromide, photographed under ultraviolet light and analyzed by Gel-Pro Analyzer 4.0. The expression of *survivin* mRNA was normalized against to *β*-*actin* mRNA.

### Western blot analysis

Cells treated with ASA were washed twice with cold PBS and lysed in lysis buffer containing 50 mmol/L Tris-HCl (pH 7.5), 150 mmol/L NaCl, 1% NP-40, 0.5% sodium deoxycholate and 0.1% SDS. Total protein was extracted from the lysates after centrifugation at 10,000 rpm for 10 min, separated by 12% SDS/polyacrylamide gel and transferred electrophoretically to nitrocellulose membrane. The membrane was blocked with 5% nonfat milk at 37°C for 1 h, incubated with antibody against survivin for 1 h, and then incubated with peroxidase conjugated rabbit anti-goat antibody for 1 h. Survivin protein signals were visualized by the enhanced chemiluminescence protocol (Pierce Chemical Co., IL, USA) and by exposure to Kodak X-Omat film (Eastman Kodak, NY, USA). The membrane was stripped, re-incubated with antibody against β-actin for 1 h, and then incubated with peroxidase conjugated anti-mouse antibody for 1 h. Protein signals were analyzed by Gel-Pro Analyzer 4.0 and the expression of survivin was normalized against the corresponding β-actin expression.

### Statistical analysis

The percentage of trypan blue stained cells, cell survival rate and the expression of survivin mRNA and protein were expressed as mean±SD. ANOVA with Bonferroni posttest was used to determine the difference among 3 or more groups. The Spearman correlation analysis was performed to analyze the relationship between cell death, survival rate or apoptosis with the concentration of ASA. All the analyses were carried out with Stata version 10.0 (STATA Corporation, College Station, TX, USA) and were based on two-tailed probabilities. A *P* value of < 0.05 was considered statistically significant.

## RESULTS

### Trypan blue exclusion

**Fig. 1:**
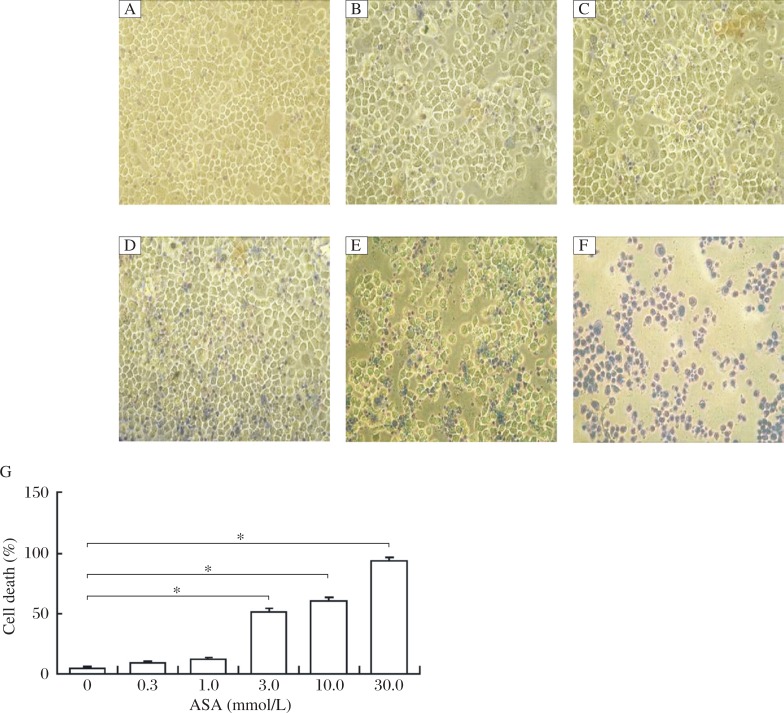
Trypan blue-stained SGC7901 cells treated with ASA for 24 h. The morphological features of cells treated with different concentrations of ASA (A: 0 mmol/L; B: 0.3 mmol/L; C: 1.0 mmol/L; D: 3.0 mmol/L; E: 10.0 mmol/L; F: 30.0 mmol/L) were analyzed by a phase-contrast microscope (200×). The percentage of cell death determined by the trypan blue exclusion assay. Data are presented as mean±SD, *n* = 3, *P < 0.05 *vs* the control group (ASA=0 mmol/L).

After treatment with ASA (0.3, 1.0, 3.0, 10.0, and 30.0 mmol/L) for 24 h, cells were incubated with trypan blue. The dead cells were stained while viable cells excluded the dye. As shown in ***Fig. 1***, there was no difference in the percentage of stained cells between the 0.3 or 1.0 mmol/L ASA group and the control group. Treatment with 3 or 10 mmol/L ASA demonstrated a cell death rate of about 50%. Furthermore, 30 mmol/L ASA resulted in a death rate of more than 90%. The Spearman correlation analysis revealed a correlation coefficient of 98.13% between cell death (%) and ASA (mmol/L) (*P* < 0.001). According to the preliminary results, the concentrations of 1, 3.0 and 10.0 mmol/L were used in the following experiments.

### Effects of ASA on SGC7901 cell viability

**Fig. 2:**
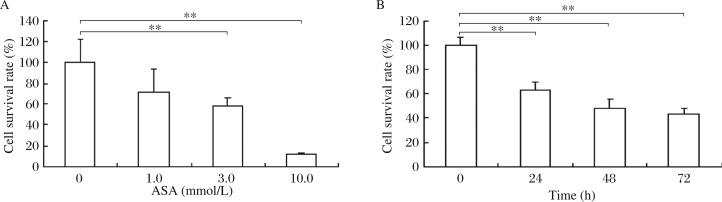
Viability assay of SGC7901 cells treated with different concentrations of ASA for 24 h (A) and 3.0 mmol/L ASA for 24 to 72 h (B) by the MTT method. Data are presented as mean ± SD, *n* = 6, **P < 0.01 *vs* the 0 mmol/L ASA or 0 h group.

SGC7901 cells were treated with various concentrations of ASA for 24 h and 3.0 mmol/L ASA for 24 to 78 h. Cell viability was determined by the MTT assay and expressed by survival rate. The results showed that 3.0 and 10.0 mmol/L ASA for 24 h decreased the survival rate by 44.6% and 88.5%, respectively, compared with the control group (***Fig. 2***). ASA at 3.0 mmol/L inhibited SGC7901 cell viability in a time-dependent manner. The survival rate was about 45% at 72 h post treatment. The Spearman correlation analysis revealed a correlation coefficient of 88.53% between apoptosis rate (%) with ASA (mmol/L) (*P* < 0.001), and 92.84% with incubation time (h) (*P* < 0.001).

### ASA induces SGC7901 apoptosis

**Fig. 3:**
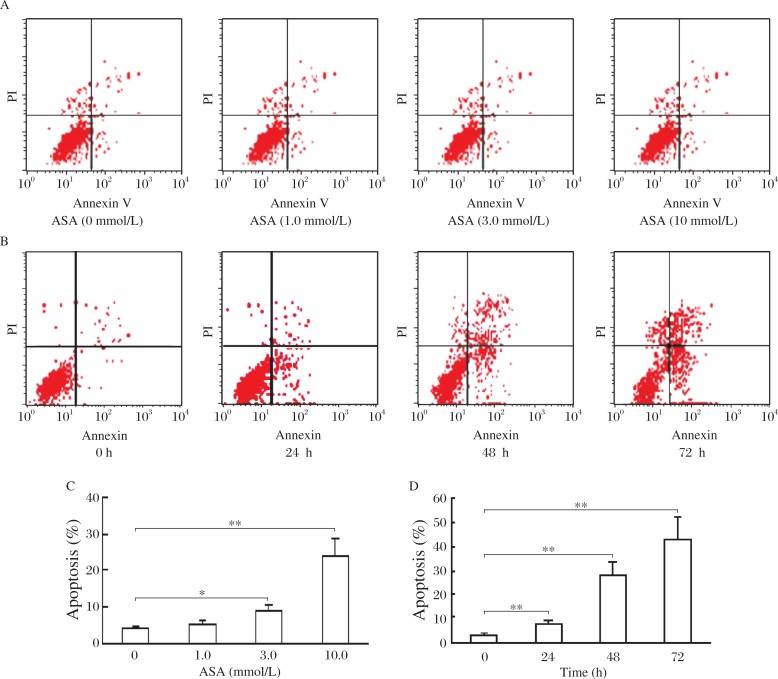
The apoptotic rate was determined by flow cytometry in SGC7901 cell lines treated with 1.0, 3.0 and 10 mmol/L ASA for 24 h (A,C) and 3.0 mmol/L ASA for 24 h 48 h and 72 h (B,D), respectively. Data are presented as mean±SD, *n* = 3. *P < 0.05, **P < 0.01 *vs* 0 mmol/L ASA or 0 h group.

The apoptosis induction of ASA on SGC7901 was determined by flow cytometry. ASA at 3.0 and 10.0 mmol/L could significantly induce SGC7901 apoptosis at a rate of 8.66% and 23.94%, respectively (***Fig. 3A*** and ***3B***). ASA also showed a time-dependent induction of apoptosis (***Fig. 3C*** and ***3D***). The apoptosis rate was 7.29%, 28.03% and 43.12%, respectively, for cells preincubated with 3.0 mmol/L ASA for 24, 48 and 72 h, while the rate was 2.76% for 0 h. The Spearman correlation analysis revealed a correlation coefficient of 92.84% between apoptosis rate (%) with ASA (mmol/L) (*P* < 0.001), and 97.16% with incubation time (h) (*P* < 0.001).

### Effects of ASA on *survivin* mRNA expression

**Fig. 4:**
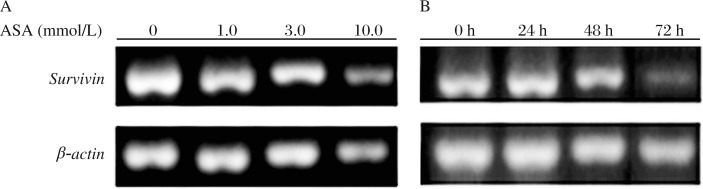
RT-PCR analysis of the mRNA expression of survivin in SGC7901 cells treated with different concentrations of ASA for 24 h (A) and 3.0 mmol/L ASA for 24, 48 and 72 h (B).

*Survivin* mRNA expression was examined by RT-PCR. Although ASA at 1.0, 3.0 and 10.0 mmol/L for 24 h all decreased the mRNA transcript levels of survivin (***Fig. 4A***), the significance was only found in the 3.0 and 10.0 mmol/L groups. After treatment with 3 mmol/L ASA for 24 to 72 h, SGC7901 cells demonstrated a time-dependent decrease in the mRNA transcript levels of *survivin* (***Fig. 4B***).

### Effects of ASA on survivin protein expression

**Fig. 5:**
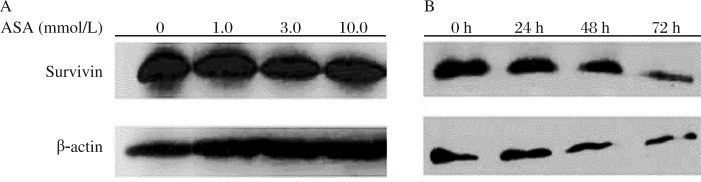
Western blotting analysis of survivin in SGC7901 cells treated with different concentrations of ASA for 24h (A) and 3.0 mmol/L ASA for 24, 48 and 72 h (B).

The survivin protein expression was determined by Western blot analysis. The results were similar to *survivin* mRNA expression. ASA at 3.0 and 10.0 mmol/L for 24 h induced a significantly lower expression of survivin protein (***Fig. 5A***). When cells were treated with 3.0 mmol/L ASA for 24 to72 h, the protein expression of survivin was decreased with the incubation time (***Fig. 5B***).

## DISCUSSION

In the present study, our results indicated that ASA could inhibit the proliferation of SGC7901 cells in a time-dependent manner. This inhibitory effect may be related to apoptosis induction. The mRNA and protein expression of apoptosis inhibitor survivin was also time-dependently reduced by ASA. The results suggest that the decrease of survivin expression by ASA may be a pivotal factor to promote apoptosis and inhibit the proliferation of SGC7901 cells.

ASA has been commonly used in the clinic for treating inflammation, mild to moderate pain, and fever. In the past two decades, a growing amount of literature has documented the anticancer effect of NSAIDs[17],[18]. Recently, large clinical studies have provided evidence of a protective effect of ASA against malignancies, such as Hodgkin lymphoma[19] and colorectal cancer[20]. Regular ASA use after the diagnosis of colorectal cancer has also been shown to be associated with a lower risk of colorectal cancer-specific and overall mortality[21]. As to gastric cancer, a meta-analysis reported an association of rational use of ASA with reduced risk of noncardia gastric cancer[22]. However, the precise mechanism has not been fully elucidated.

Previous studies indicated that the decreased synthesis of PGE2 resulted from inhibition of COX activity by NSAIDs was due to apoptosis of cancer cells[1],[4]. But recent studies provided evidence of COX-independent apoptosis induced by NSAIDs. Grösch *et al.*[3] observed that celecoxib induced apoptosis independently of the COX-2 expression in colon cancer cells. Celecoxib was found to reduce the proliferation of HCT-15 (COX-2 deficient) colon cancer xenografts in nude mice, but had no significant effect on HT-29 tumors, which express COX-2 constitutively[3]. Similar results were also demonstrated in COX-null embryofibroblasts[5]. Thus, induction of apoptosis by NSAIDs may not be restricted to COX over-expressing tumors and COX-2 independent mechanisms also have been explored by researchers. A mechanism of the disruption of mitochondrial membrane potential activating caspase 9 and downstream caspase 3 and 8 was suggested to be attributed to apoptosis induction in human oral cancer lines by celecoxib derivatives[6]. Literature on the evidence also indicated that ASA caused apoptosis via down-regulation of IL-6-dependent PGE2 or PGE2 STAT3 signaling[23], and alteration of the Mcl-1/ Noxa balance[24]. It is clear that multiple pathways with COX-dependent and -independent mechanisms are included in ASA or other NSAIDs induced apoptosis[25]. Down-regulation of anti apoptotic proteins is one of the COX-independent pathways.

ASA has been shown to downregulate anti-apoptotic protein Bcl-2 expression in human prostate adenocarcinoma LNCaP and human colorectal carcinoma CX-1 cells[26]. The down-regulation could promote the release of cytochrome c, further activate caspase 9 and 3, and induce apoptosis. Survivin is a member of the IAP family, and also has been reported to be down-regulated by ASA in hormone-refractory prostate cancer cells and transformed breast epithelial cells[14],[15]. This down-regulation was reported to be due to the inhibition of E2F-1 binding to the survivin promoter region[14]. In the present study performed with gastric cancer cells, both 3.0 and 10.0 mmol/L ASA showed an effect of down-regulating survivin expression and a time-dependent effect was demonstrated. Our findings indicated that suppression of survivin expression may be associated with the inhibition of proliferation of SGC7901 by ASA.

It has been confirmed survivin could be involved in the occurrence, progression and resistance to chemotherapy and radiotherapy of cancers[8]–[13]. By down-regulation of survivin, ASA may display an anticancer effect and may sensitize cancer cells to chemotherapy and radiotherapy. Previous studies have suggested that survivin is involved in the occurrence and development of gastric cancerous invasion and/or metastasis[27]–[29], and should be a target of chemotherapy for gastric cancer. Our results showed that ASA reduced survivin expression in SGC 7901 cells and such down-regulation may account for the anticancer effect of ASA shown by epidemiological studies. Additionally, preclinical studies have found that ASA could promote TRAIL-induced apoptosis in prostate cancer cells and breast cancer cells by survivin depletion[14],[15]. *In vivo*, ASA could work synergistically with TRAIL to reduce tumor burden in an orthotopic breast cancer xenograft model[15]. All the above findings support that ASA may be a promising sensitizer of cancer to TRAIL-based therapy. With respect to gastric cancer, survivin expression may be pivotal in resistance to chemotherapy. Overexpression of survivin protected MKN45 cells from cis-diamminedichloroplatinum induced apoptosis[30]. Down-regulation of survivin has been reported to account for the enhanced antitumor effect of paclitaxel combined with oxaliplatin[31]. Given the suppression by survivin of gastric cancer cells we found in this study, ASA could be considered a useful sensitizer of gastric cancer to chemotherapeutic drugs, such as cis-diamminedichloroplatinum. Our subsequent study will focus on explorating this area.

In summary, our results demonstrated that ASA could inhibit the proliferation of SGC7901 by suppression of *survivin* mRNA and protein expression. Considering the importance of survivin on the occurrence, progression and resistance to chemotherapy of cancers, ASA may show an anticancer effect and may be a promising sensitizer of gastric cancer to chemotherapeutic drugs. Further studies are needed to evaluate the therapeutic utility and elucidate the mechanism of ASA combined with anticancer drugs for gastric cancer.
